# The Effect of Photoperiod on Necrosis Development, Photosynthetic Efficiency and ‘Green Islands’ Formation in *Brassica juncea* Infected with *Alternaria brassicicola*

**DOI:** 10.3390/ijms22168435

**Published:** 2021-08-05

**Authors:** Violetta Katarzyna Macioszek, Mirosław Sobczak, Andrzej Skoczowski, Jakub Oliwa, Sylwia Michlewska, Magdalena Gapińska, Iwona Ciereszko, Andrzej Kiejstut Kononowicz

**Affiliations:** 1 Laboratory of Plant Physiology, Department of Biology and Plant Ecology, Faculty of Biology, University of Bialystok, 15-245 Bialystok, Poland; icier@uwb.edu.pl; 2 Department of Botany, Institute of Biology, Warsaw University of Life Sciences—SGGW, 02-787 Warsaw, Poland; miroslaw_sobczak@sggw.edu.pl; 3 Institute of Biology, Pedagogical University of Krakow, 30-084 Krakow, Poland; andrzej.skoczowski@up.krakow.pl (A.S.); jakub.oliwa@gmail.com (J.O.); 4 Laboratory of Microscopy Imaging and Specialized Biological Techniques, Faculty of Biology and Environmental Protection, University of Lodz, 90-237 Lodz, Poland; sylwia.michlewska@biol.uni.lodz.pl (S.M.); magdalena.gapinska@biol.uni.lodz.pl (M.G.); 5 Department of Plant Ecophysiology, Faculty of Biology and Environmental Protection, University of Lodz, 90-237 Lodz, Poland; andrzej.kononowicz@biol.uni.lodz.pl

**Keywords:** biotic stress, continuous light, chlorophyll *a* fluorescence, chloroplast ultrastructure, carotenoids, ‘green islands’, photoperiod

## Abstract

The main goal of growing plants under various photoperiods is to optimize photosynthesis for using the effect of day length that often acts on plants in combination with biotic and/or abiotic stresses. In this study, *Brassica juncea* plants were grown under four different day-length regimes, namely., 8 h day/16 h night, 12 h day/12 h night, 16 h day/8 h night, and continuous light, and were infected with a necrotrophic fungus *Alternaria brassicicola*. The development of necroses on *B. juncea* leaves was strongly influenced by leaf position and day length. The largest necroses were formed on plants grown under a 16 h day/8 h night photoperiod at 72 h post-inoculation (hpi). The implemented day-length regimes had a great impact on leaf morphology in response to *A. brassicicola* infection. They also influenced the chlorophyll and carotenoid contents and photosynthesis efficiency. Both the 1st (the oldest) and 3rd infected leaves showed significantly higher minimal fluorescence (F_0_) compared to the control leaves. Significantly lower values of other investigated chlorophyll *a* fluorescence parameters, e.g., maximum quantum yield of photosystem II (F_v_/F_m_) and non-photochemical quenching (NPQ), were observed in both infected leaves compared to the control, especially at 72 hpi. The oldest infected leaf, of approximately 30% of the *B. juncea* plants, grown under long-day and continuous light conditions showed a ‘green island’ phenotype in the form of a green ring surrounding an area of necrosis at 48 hpi. This phenomenon was also reflected in changes in the chloroplast’s ultrastructure and accelerated senescence (yellowing) in the form of expanding chlorosis. Further research should investigate the mechanism and physiological aspects of ‘green islands’ formation in this pathosystem.

## 1. Introduction

Modulation of light quality and quantity (photoperiod) to optimize growing conditions of plants and to investigate their influence on photosynthetic efficiency with the addition of biotic and/or abiotic stresses has become a modern approach to plant science [1,2,3]. Photoperiod influences plant development and flowering, so that short-day, long-day, and neutral-day (insensitive to photoperiod) plant species can be distinguished. Plants sense the length of the light period in leaves and spread the signal throughout the body, adjusting their physiology and development [4,5]. However, the effect of photoperiod on plant growth depends also on many factors, such as light quality and intensity, plant species and/or cultivar, and growing conditions (soil, watering, and temperature) [6,7,8]. A prolonged light period can induce stress reactions in plants, and it was recently described as a novel type of abiotic stress [9,10,11]. Moreover, the application of an additional biotic or abiotic stress to plants grown under a prolonged day results in the expression of physiological and genetic responses, e.g., activation of defense pathways [3,7,12]. Interestingly, various traits have been investigated in several plant species grown under continuous light (CL), which can have a contrasting impact on the circadian clock, photosynthesis and productivity. Growing plants under continuous light can promote their growth or it may injure them [13,14]. For example, wild tomato cultivars are insensitive to CL treatment, increasing their biomass, whereas leaf injury in the form of chlorosis, reduction in photosynthesis efficiency, and increased antioxidant enzyme activity occur in cultivated tomato cultivars grown under CL [15]. In lettuce, which is less sensitive to CL than tomato, photooxidative stress induced by CL caused enhanced production of reactive oxygen species (ROS) and lipid peroxidation, but biomass gain was also observed [16]. Recent research has revealed that exposing broccoli to continuous white light delays its yellowing and chlorophyll degradation during postharvest storage [17]. Thus, manipulating day length can be used as a new tool of modern agriculture, especially in controlled-environment plant factories [14].

Indian mustard (*Brassica juncea*) is an important crop species of the Brassicaceae family. Mostly, *B. juncea* is cultivated in Asia, Europe, and Australia for its seeds, which are used as a source of edible oil. Many cultivars of Indian mustard are also used as green vegetables [18,19]. However, *B. juncea* is exposed to many diseases, including fungus-induced ones, such as black spot disease, caused by the necrotrophic fungus *Alternaria brassicicola*, or *Alternaria* blight caused in many cases by a mixture of both *A. brassicae* and *A. brassicicola* [19,20,21]. Mustard cultivars show various degrees of susceptibility to *A. brassicicola*, such as the other brassicas, and there is a lack of known fully resistant cultivars [22,23]. *A. brassicicola* induces progressive necroses in *B. juncea* foliar tissues, preferentially infecting older leaves [19]. Spreading cell death, in this case, is mediated by the production of ROS and is associated with an increased level of salicylic acid (SA) and expression of SA-responsive gene encoding PR-1 protein (pathogenesis-related 1) [24,25]. Additionally, a reduced maximum quantum yield of photosystem II (F_v_/F_m_) and decreased non-photochemical quenching (NPQ) in infected *B. juncea* were observed [19]. 

Biotic stress often induces a plethora of various symptoms visible in the foliar tissues of plants. One such phenomenon is ‘green island’ (GI), which has been observed and described during the infection of plants by viruses, bacteria, fungi, and insects [26]. ‘Green island’ is formed at or around an infection site in the form of a greenish ring of tissues. For biotrophic and hemibiotrophic fungi in the biotrophic phase of their life cycle, GIs may aim to keep the host’s tissues alive as long as possible either by fungus-secreting cytokinins or by the accumulation of plant cytokinins at the site of infection [26,27]. However, in the case of necrotrophic fungi that actively kill host cells by secreting phytotoxins, the phenomenon of GIs is more difficult to explain. The mechanism of GI formation is not fully understood, both in terms of the factors causing them and the role they play during the infection and colonization of plants by pathogens and insects [26,27,28].

In this study, the influence of day length on the development of black spot disease in susceptible *B. juncea* plants was investigated. The current study focused on leaf position-dependent changes in photosynthesis efficiency as well as the chlorophyll and carotenoid content in infected plants. Moreover, analyses of the leaf morphology, anatomy, and chloroplast ultrastructure of the GI phenomenon observed during *A. brassicicola* infection in plants grown under a long-day photoperiod (16 h day/8 h night) and continuous light were performed.

## 2. Results

*B. juncea* plants were grown under various day-length regimes: 8 h day/16 h night, 12 h day/12 h night, 16 h day/8 h night, and CL (24 h day/0 h night), from their germination till the four-leaf stage. The plants were inoculated with *A. brassicicola* conidial suspension on the 28th day of the growing period and then were incubated for up to three days thereafter, simultaneously.

### 2.1. Leaf Size under Different Day Regimes

**Figure 1:**
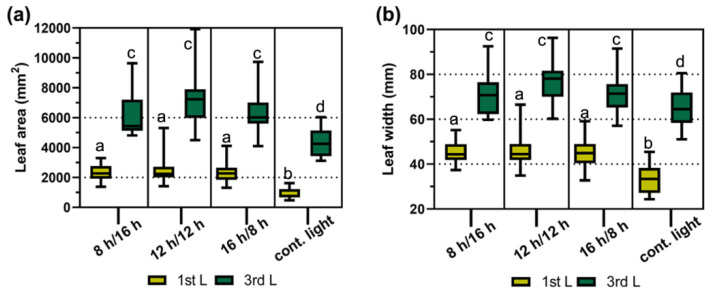
Influence of day length on the size of the 1st and 3rd leaf of *B. juncea* plants. (**a**) Leaf area; (**b**) leaf width. Plants were grown under different day-length regimes simultaneously. The columns represent the mean values ± min./max. obtained from measurements of 20–24 leaves per photoperiod (*n* = 20–24). Different letters indicate a significant difference between the means according to Duncan’s test (*p* < 0.05). Abbreviations: L, leaf; 8 h/16 h, 8 h day/16 h night; 12 h/12 h, 12 h day/12 h night; 16 h/8 h, 16 h day/8 h night; cont. light, continuous light (24 h day/0 h night).

*B. juncea* plants grown under various day-length regimes displayed differences in the selected morphological and developmental features. The plants grown under short-day photoperiods (8 h day/16 h night and 12 h day/12 h night) had shorter stems (Figure S1) and entered the flowering stage at least a week later than the ones grown under long-day regimes (16 h day/8 h night and CL). However, both the 1st (the oldest) and 3rd leaf had a significantly smaller area (*p* < 0.05) and width (*p* < 0.05) only in plants grown under CL compared to plants grown under the other three photoperiods. Continuous illumination led to a reduction in the 1st leaf area and width by approximately 53% and 26%, respectively, in comparison to leaves kept under other light regimes, whereas the leaf blade area and width of the 3rd leaf decreased on average by 30% and 9%, respectively (Figure 1).

### 2.2. Necrosis Development under Different Day Lengths

**Figure 2:**
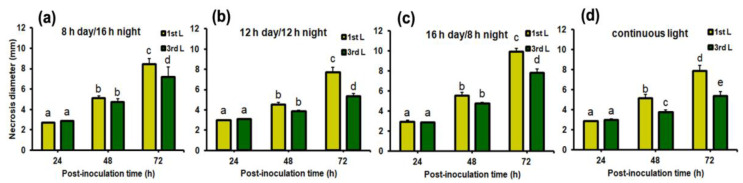
Necrosis development on the 1st and 3rd leaf of *A. brassicicola*-infected *B. juncea* plants grown under different day-length regimes: (**a**) 8 h day/16 h night; (**b**) 12 h day/12 h night; (**c**) 16 h day/8 h night; and (**d**) continuous light. The mean values ± SE were obtained in four independent experiments (*n* = 4). Different letters indicate a significant difference between the means within a photoperiod according to a post-hoc Duncan’s test (*p* < 0.05). Abbreviations: 1st L, first leaf; 3rd L, third leaf.

**Figure 3:**
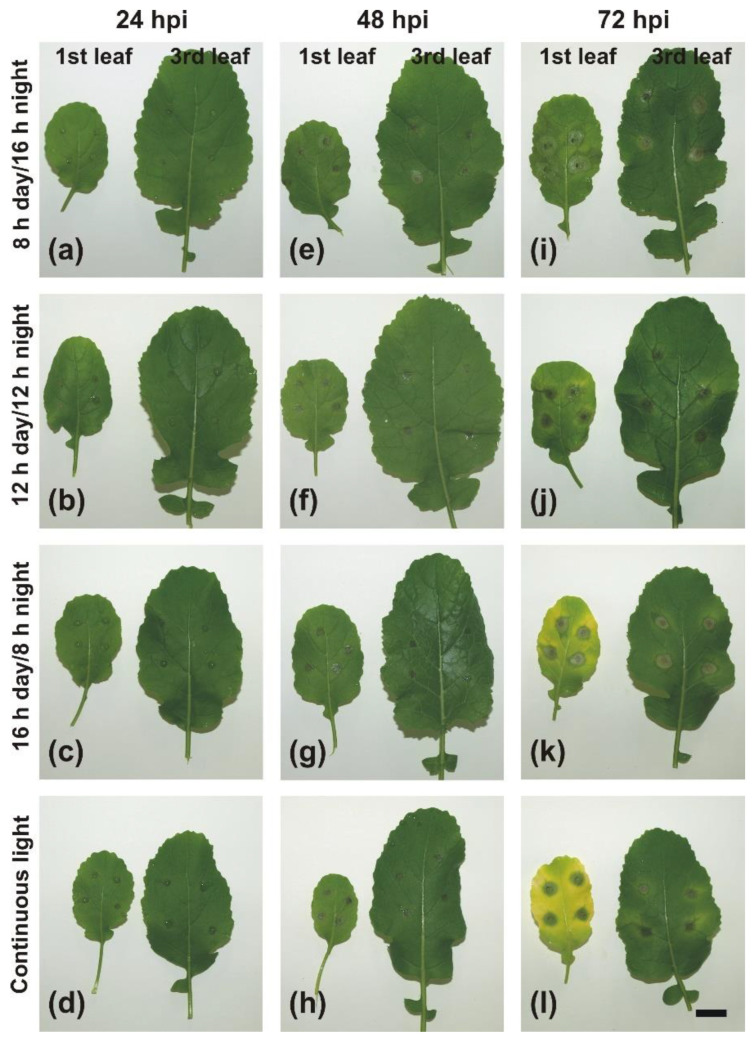
Time-course of the phenotypic response of the 1st and 3rd *B. juncea* leaf to *A. brassicicola* infection. Plants were grown and infected under different day-length regimes, simultaneously: (**a**,**e**,**i**) 8 h day/16 h night; (**b**,**f**,**j**) 12 h day/12 h night; (**c**,**g**,**k**) 16 h day/8 h night; and (**d**,**h**,**l**) continuous light, examined 24 (**a**–**d**), 48 (**e**–**h**), and 72 (**i**–**l**) hours post-inoculation (hpi). The presented images were taken in a single experiment. The 1st leaf is the oldest. Scale bar = 20 mm.

At 24 h post-inoculation (hpi), the small necrotic spots at the inoculation sites were formed on *B. juncea* leaves and they were similar in size and appearance in all implemented day-length regimes (Figure 2 and Figure 3a–d). No other changes in phenotypic response became visible on the 1st and 3rd leaf upon *A. brassicicola* infection at this time point. At 48 hpi, in general, necrosis diameters on the 1st and 3rd leaves were larger compared to 24 hpi (Figure 2). There was no difference in necrosis size between the 1st and 3rd leaf at 48 hpi except the only significant difference in necrosis diameter between the 1st and 3rd leaf found in plants grown under CL (Figure 2). On the 1st and 3rd leaf of plants grown under short-day photoperiods (8 h day/16 h night and 12 h day/12 h night), necrotic regions surrounding the inoculation sites in the form of a lighter ‘halo’ were formed (Figure 3e,f). In contrast, a necrotic ‘halo’ around the inoculation sites was absent in plants grown under a 16 h day/8 h night photoperiod or CL (Figure 3g,h).

The diameter of necrosis expanded at 72 hpi and was significantly larger on the 1st leaf than on the 3rd one in plants grown under all examined day-length regimes (*p* < 0.05). The largest necroses on both the 1st and 3rd leaf were formed in plants grown under a 16 h day/8 h night photoperiod (9.95 ± 0.3 and 7.8 ± 0.38, respectively), while the smallest ones were developed on leaves of plants grown under 12 h day/12 h night (7.6 ± 0.47 and 5.35 ± 0.28, respectively) and CL (7.8 ± 0.5 and 5.39 ± 0.39, respectively) (Figure 2). Differences in chlorosis extent and intensity became evident on both the 1st and 3rd leaf of plants grown under all the examined day-length regimes at 72 hpi (Figure 3i–l). Under short-day photoperiods, the inoculation sites were surrounded by rings of necrotized cells and both the 1st and 3rd leaf still remained greenish (Figure 3i,j). In contrast, under long-day regimes (16 h day/8 h night or CL) the 1st leaf turned yellowish, whereas on the 3rd leaf only a discrete chlorotic ring formed around the necrosis. The chlorosis proceeded outwardly from infection sites. On almost 25% of infected plants, the region of necrotized infection sites on the 1st leaf was surrounded by a strongly green-stained ring (‘green island’) forming a ‘halo’ around the infection sites, whereas almost the whole leaf blade turned yellow (Figure 3k,l). The 3rd leaf was generally green at this time point, but necrotized inoculation sites were surrounded by a lighter ring of cells and outwardly embedded in a yellowish tissue. However, the depth of leaf discoloration was more obvious in leaves of plants kept under CL (Figure 3l).

Factorial analysis of variance revealed that the necrosis diameter was significantly influenced by day length (photoperiod, F = 12.274, *p* < 0.001), leaf position (F = 41.573, *p* < 0.001), and post-inoculation time (F = 348.569, *p* < 0.001).

### 2.3. Changes in Chlorophyll and Carotenoid Contents

Analysis of control leaves of *B. juncea* plants revealed that the 3rd leaf contained on average more photosynthetic pigments than the 1st one (the oldest). Moreover, the highest chlorophyll and carotenoid contents were observed in the 1st leaf of control plants grown under a 16 h day/8 h night photoperiod and the lowest levels of pigments were found in plants grown under CL (Figure S2). In the 3rd control leaf, higher amounts of photosynthetic pigments were detected in plants grown under long-day regimes. Ratios of chlorophyll *a*:*b* and total chlorophyll:carotenoids were similar in both leaves of plants grown under all the implemented day-length regimes (Figure S2).

**Figure 4:**
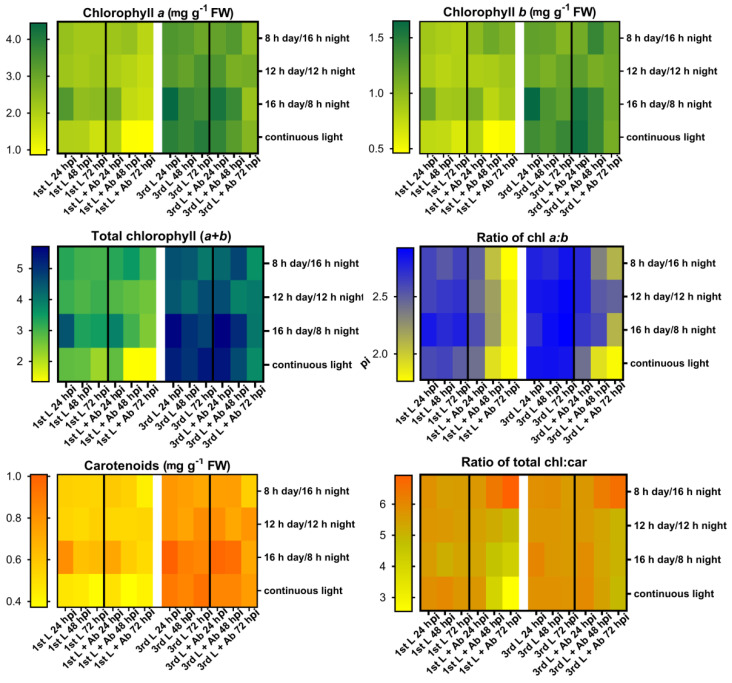
Heatmaps based on the mean values of chlorophylls and carotenoids contents in the 1st and 3rd leaf of *B. juncea* plants during the progress of *A. brassicicola* infection. The experiment was performed in plants grown under different day-length regimes and was independently repeated four times (*n* = 4). Details of the statistical analysis of the parameters are shown in the Supplementary Material Tables S1 and S2, and Figure S2. Abbreviations: 1st L, first leaf; 3rd L, third leaf; Ab, *A. brassicicola*; car, carotenoids; chl, chlorophyll; FW, fresh weight.

At 24 hpi, in both (the 1st and 3rd) leaves of plants grown under all implemented light regimes, contents of chlorophyll a (chl *a*), chlorophyll *b* (chl *b*), total chlorophyll (chl *a* + *b*), carotenoids (car), ratios of chlorophyll *a*:*b* (chl *a*:*b*), and total chlorophyll:carotenoids (total chl:car) were similar in control and infected leaves, regardless of the appearance of small necrotic spots (Figure 4, Table S1). In general, the content of chlorophylls, carotenoids, and calculated values of pigments ratios were highest in control and infected leaves of plants grown under a 16 h day/8 h night photoperiod, and the lowest values were noted for leaves of plants grown under CL. The photosynthetic pigment contents in plants grown under short-day photoperiods (8 h day/16 h night and 12 h day/12 h night) ranged between values obtained for a 16 h day/8 h night photoperiod and CL. It has to be emphasized that the contents of chl *a*, chl *b*, and car as well as values of ratios of chl *a*:*b* and total chl:car were higher in the 3rd (younger) leaf than in the 1st one in control and infected plants (Figure 4, Table S1).

The ratio of chl *a*:*b* decreased in the 1st and 3rd leaf of infected plants at 48 hpi compared to the uninfected control plants grown under all examined day-length regimes, but the total chl and car contents in infected leaves were at similar levels as in the control samples. However, the chl *a* content decreased significantly only in the 1st leaf of plants grown under a 16 h day/8 h night and CL and in the 3rd leaf of plants grown under a 12 h day/12 h night and 16 h day/8 h night. A significant decrease in both chl *a* and *b* contents only occurred in the 1st leaf of infected plants grown under CL compared to the control plants (Figure 4, Table S1). Carotenoid content was at a similar level in both investigated infected leaves (1st and 3rd) when compared to the control leaves in plants grown under all implemented photoperiods and CL, regardless of large necroses observed at this time point of post-inoculation (Figure 2 and Figure 4).

Content of chl *a* decreased significantly in both infected leaves compared to the control at 72 hpi in plants grown under all investigated day-length regimes, except plants grown under an 8 h day/16 h night, where the chl *a* and chl *b* contents were at similar levels in the control and infected leaves at this time point of infection. However, the amount of total chl and the ratio of total chl:car decreased significantly in the 1st and 3rd infected leaves under all implemented day-length regimes, with the surprising exception of a significant increase in the total chl:car ratio in plants grown under an 8 h day/16 h night photoperiod (Figure 4, Table S1). Similarly to 24 and 48 hpi, the carotenoids content was similar in both infected leaves compared to the control ones at 72 hpi (Figure 4). The highest decrease in the investigated photosynthetic pigments values, including the ratio of total chl:car, was observed in the 1st leaf of plants grown under CL at this final time point of post-inoculation (Figure 4, Table S1). 

Factorial analysis of variance revealed that the contents and ratios of chlorophylls and carotenoids were significantly influenced by day length (photoperiod), treatment (control/infection), leaf position, and post-inoculation time (*p* < 0.001; Table S2). Only the content of chl *b* was not significantly influenced by treatment (F = 0.31, *p* = 0.58).

### 2.4. Analysis of Chlorophyll a Fluorescence

Control plants grown under CL showed significantly higher minimum fluorescence (F_0_) and reduced photosystem II (PSII) maximum quantum yield (F_v_/F_m_ < 0.7) compared to control plants grown under other investigated day-length regimes (Figure S3). This relationship was the most evident in older leaves (the 1st), although in the 3rd leaf of plants grown under a 16 h day/8 h night photoperiod also had a decrease in F_v_/F_m_ value. The other chlorophyll *a* fluorescence parameters, such as plant viability (Rfd), non-photochemical quenching (NPQ), photochemical quenching (qP), and the fraction of PSII centers that are ‘open’ based on the lake model of PSII steady-state in light (qL), were generally higher in the 3rd leaf than in the 1st one of plants grown under all investigated day-length regimes (Figure S3). The highest NPQ value was observed in the 1st leaf (at all time points) of control plants grown under CL, while in the 3rd leaf NPQ values were the highest in plants grown under a 16 h day/8 h night photoperiod. In control plants grown under CL, older leaves generally showed the lowest values of qP and qL. In younger leaves (the 3rd ones), this trend was not so clear (Figure S3).

**Figure 5:**
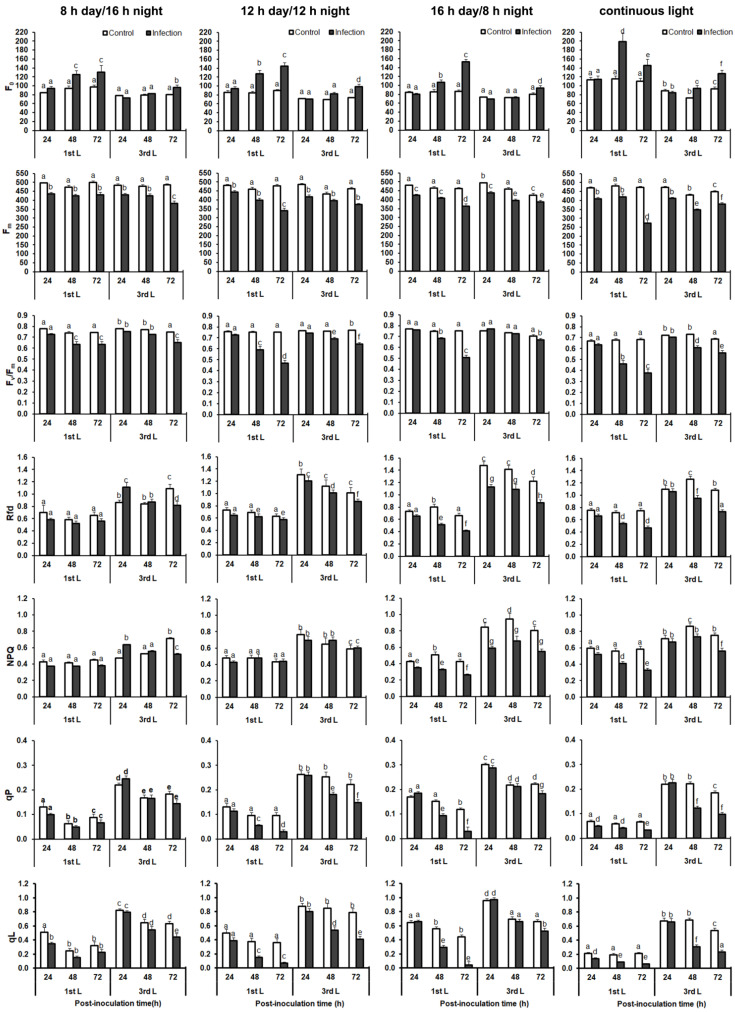
Time-course changes in chlorophyll *a* fluorescence of *B. juncea* 1st and 3rd leaves in response to *A. brassicicola* infection. Plants were grown and infected under different day-length regimes simultaneously. The mean values ± SE were obtained from three independent experiments (*n* = 3). Different letters indicate a significant difference between the means within a photoperiod according to a post-hoc Duncan’s test (*p* < 0.05). Abbreviations: 1st L, first leaf; 3rd L, third leaf; F_0_, minimum fluorescence; F_m_, maximum fluorescence; F_v_/F_m_, maximum quantum yield of PSII; NPQ, non-photochemical quenching; Rfd, fluorescence decline ratio; qP, photochemical quenching; qL, fraction of PSII open centers.

In the 1st leaf of *B. juncea* infected with *A. brassicicola* at 24 hpi, only a slight decrease in maximum fluorescence (F_m_) was observed in plants grown under examined day-length regimes and additionally a decrease in qL and qP values in infected plants grown under continuous light was observed compared to the control plants (Figure 5). At 24 hpi, the fungus caused a significant decrease in Rfd and NPQ values in the 3rd leaf of plants grown under a 16 h day/8 h night photoperiod and an increase in the values of these parameters in plants grown under an 8 h day/16 h night photoperiod was observed. Changes in the other investigated chlorophyll *a* fluorescence parameters were not statistically significant at this post-inoculation time (Table S4).

The reduction of light absorption by LHCII (increase in F_0_) did not occur until 48 hpi (Figure 5). At this time, an increase in F_0_ value was found in the 1st leaf of infected plants grown under all implemented day-length regimes compared to the control. However, the highest F_0_ value was observed in plants grown under CL. The maximum quantum yield of PSII (F_v_/F_m_), measured in the 1st leaf, decreased consistently in infected plants at 48 hpi. However, a decrease in the F_v_/F_m_ value was the greatest in plants grown under CL. At 48 hpi, a decrease in Rfd value occurred in both leaves of plants grown under a 16 h day/8 h night photoperiod and CL compared to the control. In plants grown under a 12 h day/12 h night photoperiod, a decrease in Rfd value was recorded only in the 3rd leaf, and it was not observed at all in plants grown under an 8 h day/16 h night photoperiod (Figure 5). The non-photochemical quenching value was also significantly lower in inoculated plants grown under a 16 h day/8 h night photoperiod and CL compared to the control. At 48 hpi, the qL and qP values did not change under the influence of the pathogen, only in *B. juncea* grown under an 8 h day/16 h night photoperiod, while they generally decreased in plants grown under the other day-length regimes.

At 72 hpi, an increase in F_0_ combined with a decrease in F_v_/F_m_ was already visible in infected plants compared to the control, especially in the 1st leaf (Figure 5). However, it should be emphasized that a decrease in F_v_/F_m_ was the lowest in infected plants grown under an 8 h day/16 h night photoperiod compared to plants grown under all the other day-length regimes. At 72 hpi, there was a decrease in NPQ and qP values in plants grown under all the investigated day-length regimes, except for the 1st leaf of plants grown under an 8 h day/16 h night photoperiod (Figure 5).

The chlorophyll *a* fluorescence parameters showed significant dependence on the light reactions of photosynthesis in *B. juncea* plants inoculated with *A. brassicicola* on day length (photoperiod), treatment (control/infection), leaf position, and post-inoculation time (*p* < 0.001, Table S4), and astonishingly only F_m_ was not significantly influenced by leaf position (F = 3.46, *p* = 0.063).

### 2.5. ‘Green Islands’ Formation

As mentioned above, approximately 25% of the plants grown under long-day regimes (16 h day/8 h night and CL) and inoculated on the 28th day of growth showed a ‘green island’ phenotype on the 1st infected leaves at 72 hpi. To investigate the appearance and formation of the GI phenotype, the plants were grown under both long-day regimes and four mature leaves were inoculated with *A. brassicicola* conidial suspension on the 35th day of the growing period. 

**Figure 6:**
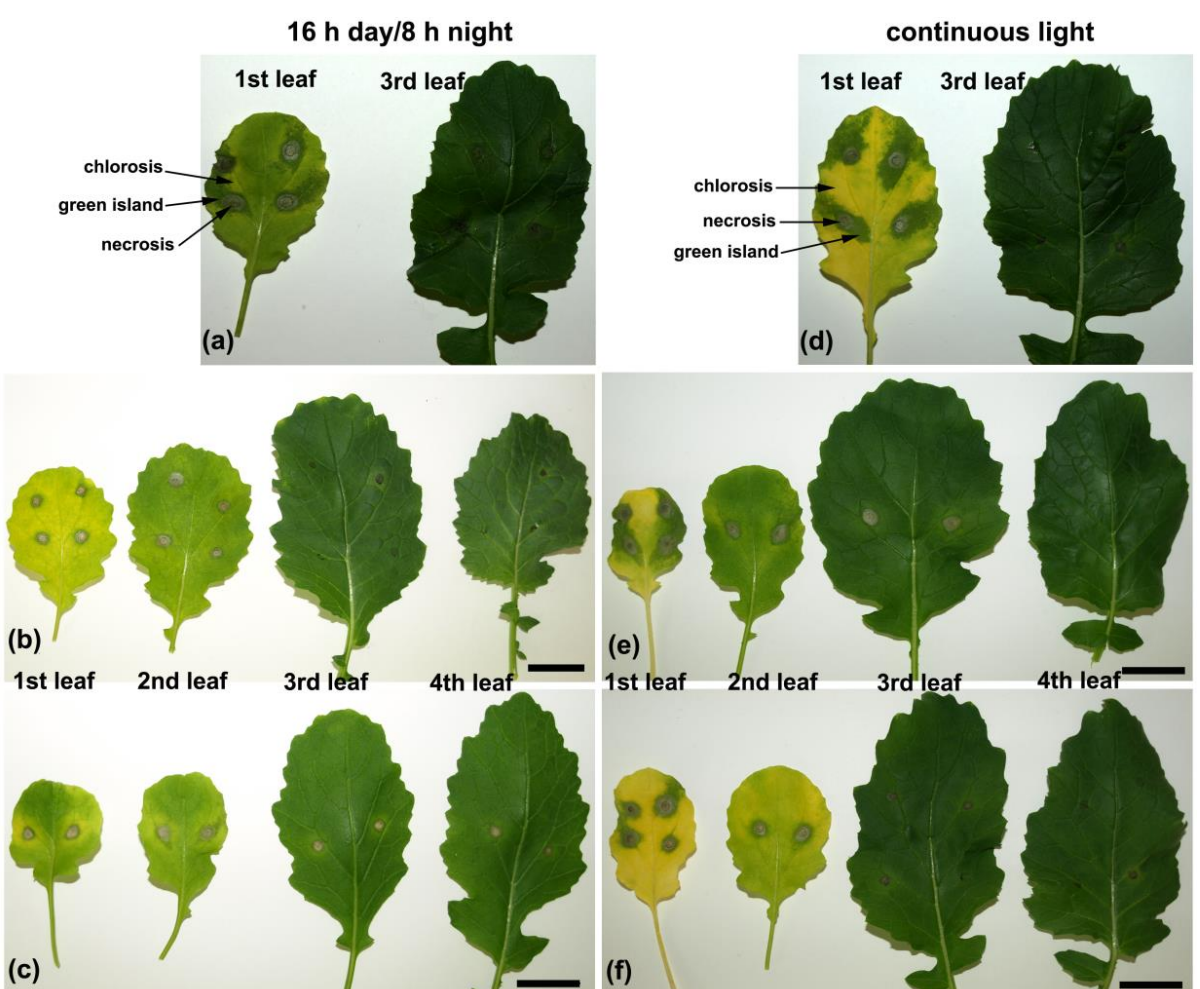
‘Green island’ phenotypes on *B. juncea* leaves infected with *A. brassicicola* at 48 hpi. Plants were grown under a 16 h day/8 h night photoperiod (**a**–**c**) and continuous light (**d**–**f**) for 35 days. Four fully developed leaves were infected. The 1st leaf is the oldest. The experiment was independently repeated twice. Scale bars = 20 mm.

Only small necrotic spots similar in size were observed in all four infected leaves at 24 hpi (Figure S4). The GI phenotype appeared on the 1st and 2nd leaves of approximately 30% of plants grown under both investigated day-length regimes already at 48 hpi (Figure 6, Figure S4). However, the GI phenotype was still absent on the 3rd and 4th infected leaves at this time of post-inoculation and only necroses surrounded by discrete rings of lighter greenish tissues were visible (Figure 6). Different morphological zones could be distinguished around the inoculation site on the 1st infected leaf (Figure 6a,d). The necrosis that appeared at the inoculation site was surrounded by a necrotic ‘halo’. More outwardly, a GI zone was formed that enclosed the necrotic region, and the other parts of the leaf blade were subjected to strong chlorosis. However, the most frequently occurring GI phenotype on the 1st leaf of plants grown under a 16 h day/8 h night photoperiod differed from the phenotype that appeared on the 1st leaves of plants grown under CL. The 1st leaves were lighter green compared to the 3rd leaves of plants grown under a 16 h day/8 h night and their necroses were surrounded by a regular ring of greenish tissues and a larger chlorotic ring of yellowish tissues was visible (Figure 6a–c). The 1st leaves of plants grown under CL were yellow and irregular GIs were formed outwardly from the necrotic region and were generally larger (Figure 6e,f) than on the 1st leaves of plants grown under a 16 h day/8 h night photoperiod (Figure 6b,c). Interestingly, the 2nd leaves of plants grown under CL showed a GI phenotype with a regular ring of greenish tissue similar to that frequently observed in the 1st leaves of plants grown under a 16 h day/8 h night photoperiod (Figure 6 b,c,e,f). 

At a later stage of infection, 72 hpi, the GI region in the form of a greenish ring was still visible as well as a distinct yellow ring of chlorosis in the 1st and 2nd leaves of plants grown under a 16 h day/8 h night photoperiod, whereas only necrotic spots with a discrete chlorotic ring were present in the 3rd and 4th leaves (Figure S4). In plants grown under CL, most of the GI zone disappeared and only a discrete ring of greenish tissue was visible on the 1st leaf. The whole 2nd leaf turned yellow with an irregular GI surrounding the necrosis, and on the 3rd leaf, a ring of GI surrounding necrosis with a clear ring of yellow tissue appeared. The 4th leaf stayed green with clear and restricted necrotic spots (Figure S4).

#### Anatomy and Ultrastructural Analysis

**Figure 7:**
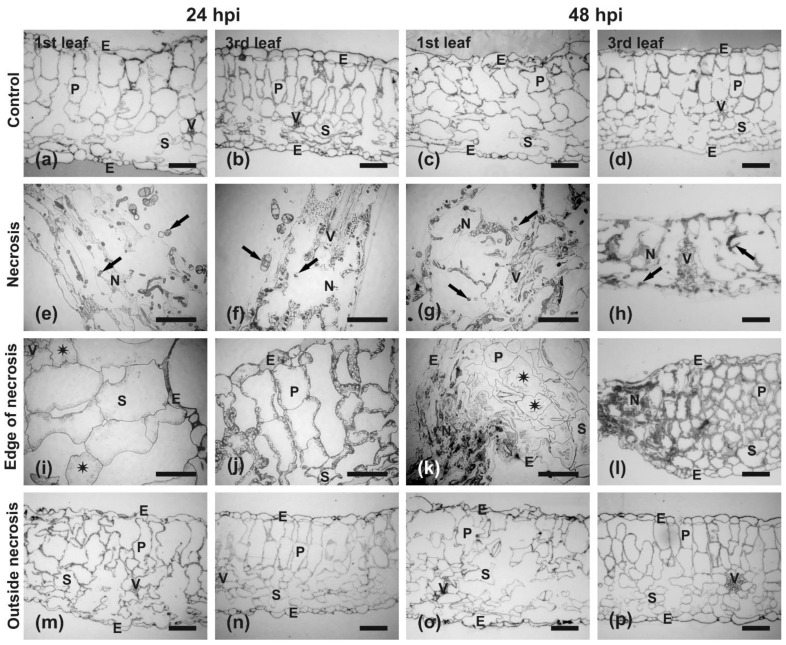
Light microscopy images showing the anatomy of different regions of *B. juncea* leaves infected with *A. brassicicola*. Plants were grown under a 16 h day/8 h night photoperiod for 35 days and inoculated with *A. brassicicola*. The samples were collected from the 1st (**a**,**c**,**e**,**g**,**i**,**k**,**m**,**o**) and 3rd (**b**,**d**,**f**,**h**,**j**,**l**,**n**,**p**) leaf at 24 (**a**,**b**,**e**,**f**,**i**,**j**,**m**,**n**) and 48 hpi (**c**,**d**,**g**,**h**,**k**,**l**,**o**,**p**). Micrographs of sections taken from uninfected control leaves (**a**–**d**); necrotized inoculation sites (**e**–**h**); edge of necrosis (**i**–**l**; **k**, ‘green island’ region); and areas outside necrosis (**m**–**p**; **o**, chlorosis). Abbreviations: E, epidermis; N, necrosis; P, palisade mesophyll; S, spongy mesophyll; V, vascular bundle. Arrows point to hyphae and asterisks indicate plasmolyzed cells. Scale bars = 10 µm.

To avoid extremes, we decided to examine and compare the anatomy and ultrastructure of different regions of the infected 1st and 3rd leaves at 24 and 48 hpi from plants grown under a 16 h day/8 h night photoperiod. Comparison of anatomical sections taken from specimens collected at 24 and 48 hpi from the 1st and 3rd control leaves showed very similar anatomical organization (Figure 7a–d). The leaves had continuous layers of upper and lower epidermis enclosing mesophyll composed of palisade parenchyma, arranged in two tiers, and spongy parenchyma. The vascular bundles were located at the interface between both types of mesophyll. The chloroplasts were clearly visible inside the mesophyll cells and were located along the cell walls (Figure 7a–d). 

In infected leaves, at the inoculation site, already at 24 hpi all mesophyll cells were destroyed and only their outlines could barely be recognized (Figure 7e,f). The same situation was also observed in this region at 48 hpi, and it clearly corresponded to necrotic lesions visible in the center of the inoculation spots (Figure 6). The degraded cells ended very abruptly at the edge of necrosis in both the 24 and 48 hpi samples (Figure 7i–l). However, mesophyll cells outside the necrosis in the 3rd leaf seemed to be filled with proliferating cytoplasm and chloroplasts (Figure 7j,l), in contrast to the 1st leaf where cytoplasm in cells surrounding the necrosis appeared to be plasmolyzed with only a small amount of the cytoplasm detaching from the cell walls (Figure 7i,k). In the region of chlorosis surrounding GI at the outer border of necrosis at 48 hpi (Figure 6), well-preserved mesophyll cells with clearly recognizable chloroplasts were present at 24 hpi (Figure 7m,n). Mesophyll cells were still nicely preserved at 48 hpi, but chloroplasts were hardly recognizable, especially in mesophyll cells of the 1st leaf (Figure 7o versus Figure 7p).

**Figure 8:**
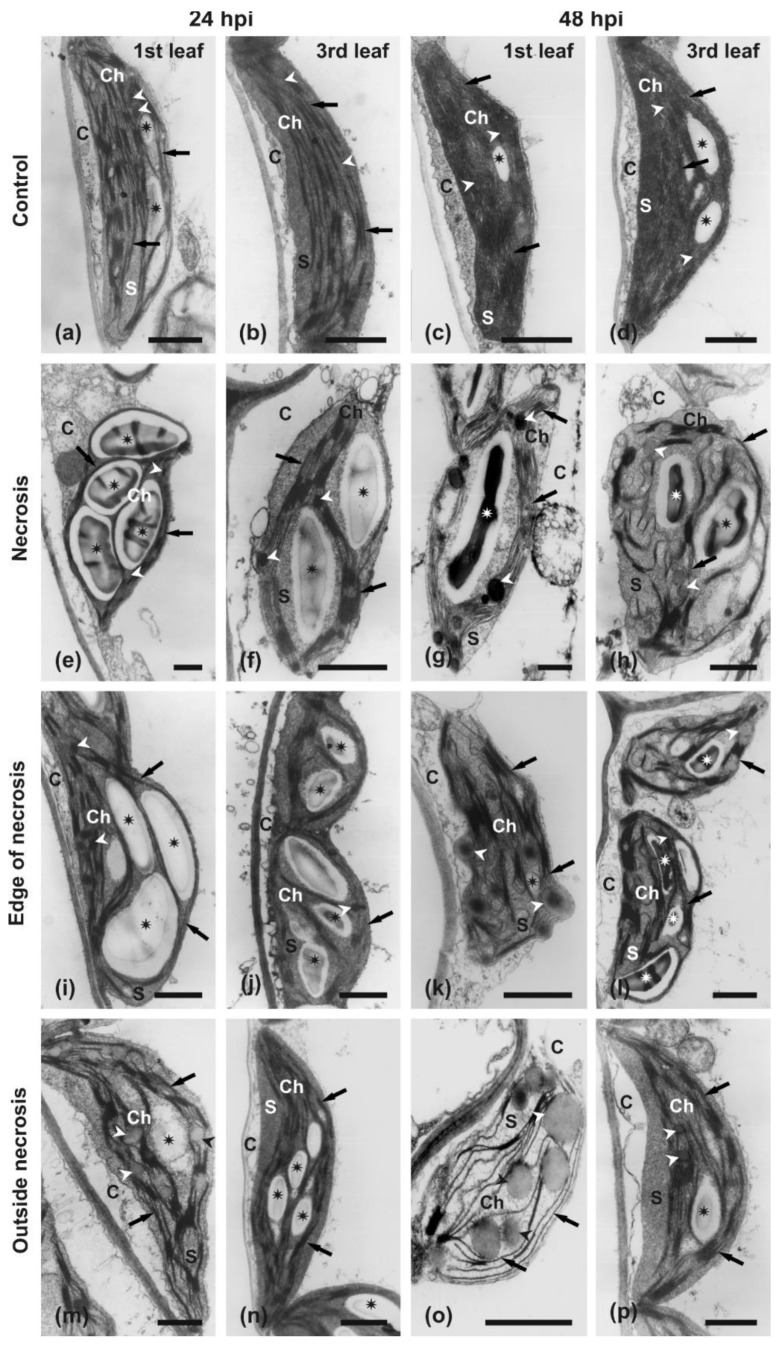
Transmission electron microscopy images showing the ultrastructure of chloroplasts in mesophyll cells from different regions of *B. juncea* leaves infected with *A. brassicicola*. Plants were grown under a 16 h day/8 h night regime for 4 weeks and inoculated with *A. brassicicola*. Samples collected from the 1st (**a**,**c**,**e**,**g**,**i**,**k**,**m**,**o**) and 3rd leaf (**b**,**d**,**f**,**h**,**j**,**l**,**n**,**p**) at 24 (**a**,**b**,**e**,**f**,**i**,**j**,**m**,**n**) and 48 hpi (**c**,**d**,**g**,**h**,**k**,**l**,**o**,**p**). Electronograms of ultrathin sections taken from control uninfected leaves (**a**–**d**), inoculation sites—necrosis (**e**–**h**), edge of necrosis (‘green island’) (**i**–**l**), and area outside of the necrosis (chlorosis) (**m**–**p**). Abbreviations: C, cytoplasm; Ch, chloroplast; S, stroma. Arrows indicate thylakoids, arrowheads point to plastoglobules, and asterisks label the starch grains. Scale bars = 1 μm.

At the ultrastructural level, the current study focused on the organization and comparison of the chloroplast ultrastructure using transmission electron microscopy (TEM). In both the 1st and 3rd control, non-inoculated leaves, chloroplasts had the typical crescent-like shapes (Figure 8a–d) and were present in the layer of well-preserved cytoplasm. Their stroma was strongly electron-dense. The thylakoid system was well-developed, and cisternae formed regular parallel arrays. Grana were abundantly formed. Chloroplasts contained large grains of transitory starch, as samples were collected at 4–6 h after commencement of the day-time period (light exposition). The number of starch grains varied between 1 and 4 per chloroplast, depending on the section level. Only a few small plastoglobuli were observed (Figure 8a–d).

Upon inoculation and formation of infection spots, the chloroplasts in central regions of the spots (necrosis) were strongly changed morphologically in the 1st and 3rd leaf already at 24 hpi (Figure 8e,f). They lost their regular outlines and turned irregular. The plastidial envelope seemed to remain intact, but the stroma turned more electron-translucent and acquired a fine granular appearance. The thylakoid system was compressed by extremely large transitory starch grains in the 1st leaf (Figure 8e). In the 3rd leaf, the arrangement of the thylakoid system was not impacted so strongly by growing starch grains, but areas of thylakoid-free stroma were present instead (Figure 8f). In both leaves, the starch grains could occupy more than half of the chloroplast section surface. At 48 hpi, chloroplasts become irregularly round in outlines (Figure 8g,h). They were apparently still surrounded by intact envelopes, but their stroma was more electron-translucent than observed at 24 hpi. Thylakoids were still present, but a few grana were present in chloroplasts collected from the 1st leaf (Figure 8g), and yet fewer were present in chloroplasts from the 3rd leaf (Figure 8h). Starch grains were still large and, additionally, numerous large plastoglobuli were formed (Figure 8g,h). The chloroplasts were present inside the cytoplasm that was deteriorating in 24 hpi samples (Figure 8e,f) and almost completely absent in samples of both leaves collected at 48 hpi (Figure 8g,h).

At the edge of the necrosis region, chloroplasts generally retained their typical shape and organization in both leaves at 24 hpi (Figure 8i,j). They used to be more round in outlines than in control samples, but their thylakoid system was well-developed and numerous grana were present. In contrast to control samples, the arrangement of thylakoids was strongly disturbed by extremely large starch grains. A similar ultrastructure of chloroplasts was also observed in samples collected from the edge of necrosis region (‘green islands’) of both leaves at 48 hpi (Figure 8k,l). However, in contrast to samples collected at 24 hpi, their outlines became more irregular, numerous plastoglobuli were formed, and the cytoplasm of GI cells was degraded (Figure 8l versus Figure 8k).

In the area outside necrosis, the chloroplasts were still crescent-shaped, but their outlines were irregularly bent in the 1st leaf at 24 hpi (Figure 8m). Their thylakoids were still well-preserved, but grana seemed to fuse. They contained large starch grains and numerous plastoglobuli. The cytoplasm of mesophyll cells was degraded and hard to recognize. At the same time, chloroplasts in mesophyll cells in the 3rd leaf still had lenticular shapes, but the thylakoid system was usually slightly curved, leaving parts of the stroma free of thylakoids (Figure 8n). The stroma was electron dense and it contained numerous large starch grains and only a few plastoglobuli. The cytoplasm was apparently deteriorating, but clearly recognizable. At 48 hpi, the chloroplasts were completely degraded in samples from the 1st leaf collected in the region outside the necrosis (chlorosis) (Figure 8o). Their outlines were usually round, but the plastidial envelope was broken. Their stroma was extremely electron-translucent, and the thylakoid system consisted of a low number of thylakoids and only a few residual grana. No starch grains were present, but numerous large plastoglobuli were formed. The cytoplasm was usually degraded, but in some cells, it was still recognizable, but strongly condensed and granular. At the same time, in the mesophyll cells of the 3rd leaf, the chloroplasts became slightly swollen and relatively large areas free of thylakoids were formed (Figure 8p). Their stroma was still uniformly electron-dense. The thylakoid system was well-preserved, and numerous grana were clearly recognizable. Starch grains and a few small plastoglobuli were still present.

## 3. Discussion

### 3.1. Differential Influence of Day Length on Leaf Morphology and Necrosis Formation

One of the recently described abiotic stresses is photoperiodic (circadian) stress caused by the extension of the light period [10]. Seedlings of *B. oleracea* var. *albogabra* (kale) grown under a prolonged light (16 h day/8 h night) show decreased height, larger cotyledons, and greater dry matter compared to sprouts grown under short-day photoperiods [29]. Mature *B. oleracea* plants subjected to continuous illumination during postharvest storage display delayed yellowing and chlorophyll degradation [17]. In this study, various day-length regimes (8 h day/16 h night, 12 h day/12 h night, 16 h day/8 h night, and 24 h day/0 h night) were implemented during a whole period of *B. juncea* growth and infection by *A. brassicicola*, which obviously affected leaf morphology and necroses formation. Interestingly, only the application of CL significantly reduced the size of both the 1st and 3rd leaf of *B. juncea* compared to the other implemented day-length regimes (Figure 1). Leaf size is an important plant trait determining photosynthesis, development, and biomass, controlled by genes and influenced, among other factors, by photoperiod [30,31]. Early flowering of *B. juncea* observed under CL (data not shown) correlated with a reduction in leaf size, which was not observed in plants grown under long-day (16 h day/8 h night) or short-day photoperiods. And most of mustard cultivars requires a long day for induction of flowering [32]. Delayed flowering time causes an increase in the leaf size of barley. Such a correlation between leaf size and flowering may be due to the allocation of nutrients to developing flowers [30].

In general, necrotrophic fungi-induced lesions of foliar tissues often develop preferentially on older leaves of susceptible cultivars, whereas younger leaves show some level of tolerance/resistance to a fungus. Such a phenomenon is also characteristic for *Brassica* species (*B. oleracea* and *B. rapa*) infected with various pathogens, such as the oomycete *Hyaloperonospora parasitica* or a typical necrotroph such as *A. brassicicola* [33,34,35]. Moreover, a previous study revealed that necrotic lesions on *B. juncea* developed in a leaf position-dependent manner during *A. brassicicola* infection—the older the leaf, the larger the necrosis [19]. The most probable factor responsible for such a difference in response of a particular *B. juncea* leaf to the necrotrophic fungus can be due to an elevated content of primary and secondary metabolites, which can restrict the growth and development of a fungal pathogen in younger leaves [19,36,37]. However, in this study, the implemented light periods differentially influenced necrosis development in *B. juncea* plants, although necrotic spots still developed in a leaf position-dependent manner. The largest necroses were observed in plants grown under a 16 h day/8 h night photoperiod, whereas the smallest lesions were noted in plants grown under CL (Figure 2 and Figure 3). The incessant light period (24 h day/0 h night) reduced disease development, and probably also inhibited the growth of the fungus. Circadian rhythm influences plant immunity, although plants seem to be more vulnerable to pathogen attack during the light period, because of photosynthetic activity and open stomata. However, a peak of jasmonic acid (JA), a hormone that is required in plant defence against necrotrophic fungi such as *A. brassicicola*, is observed in the middle of a light period in *Arabidopsis* [38]. Lack of a dark period may enhance plant resistance against *A. brassicicola* and also through extensive production of metabolites with antifungal properties, e.g., phenolic compounds or, characteristic for Brassicas, glucosinolates [39,40]. Glucosinolate biosynthesis is also regulated by light and it has been demonstrated that dark exposure decreases its content in Chinese cabbage seedlings [40,41]. Moreover, several phytopathogenic fungi, e.g., the necrotroph *Botrytis cinerea*, display a circadian rhythm, and light influences their development and stress responses [42,43]. In *Alternaria* species, growth, sporulation, and even toxins production are inhibited by a prolonged light period [44]. Thus, continuous illumination without a dark period can be a stress factor for both the host plant and pathogenic fungus.

### 3.2. Day Length-Dependent Negative Regulation of Photosynthesis

Generally, biotic stress negatively affects photosynthesis in many plant species. Infections in the form of spreading necroses and chlorosis can reduce the assimilation area of foliar tissues influencing photosynthesis and decreasing the content of photosynthetic pigments [45,46]. Necrotrophic fungi secrete phytotoxins that can directly interact with chloroplast proteins or suppress host physiology, causing extensive chlorosis and accelerated senescence [47]. In this study, chlorophyll and carotenoid contents depended on a leaf position and day length in *B. juncea* plants. The highest content of photosynthetic pigments was observed in both investigated control leaves of *B. juncea* plants grown under an optimal photoperiod of 16 h day/8 h night (Figure 4 and Figure S2). Degradation of chlorophylls (chl *a*, chl *b*, and total chl) was correlated with the progression of necrosis, although it was not noted until 48 hpi under all the examined day-length regimes (Figure 3). Reduction of the chl *a:b* ratio was mostly related to a significant decrease in chl *a* content. A similar trend of a decreased chlorophyll content has been described during progressive infection of *B. oleracea* with *A. brassicicola* [35] and *B. juncea* infected with *A. brassicae* [48]. Chlorophyll content decreased in tolerant and susceptible *B. napus* and *B. rapa* cultivars to *A. brassicicola* infection with much stronger degradation in susceptible cultivars [49]. Lowered chl *a* level accompanied by an extensive production of ROS has been also described in susceptible *B. napus* infected with *Leptosphaeria maculans*, which induces yellowish necrotic spots [50]. The appearance of spreading chlorosis followed by a decrease in chlorophyll level is also characteristic for other pathosystems of brassicas with pathogenic fungi, such as *Sclerotinia sclerotiorum* or the oomycete *Albugo candida* [22]. Surprisingly, the carotenoid content did not change significantly in *B. juncea* in response to *A. brassicicola* infection, except for little fluctuations in their level at 72 hpi, mostly in plants grown under short-day photoperiods (Figure 4). Carotenoids along with chlorophylls are important photosynthetic pigments that are components of photosystems, they absorb an excess of light, and play a role in photoprotection [51]. In tolerant and susceptible *B. napus* and *B. rapa* cultivars, a decrease in the carotenoid levels in response to *A. brassicicola* has been noted [49]. However, an increase in carotenoid content has been described in resistant *B. napus* cultivars infected with *L. maculans*, while in susceptible oilseed cultivars the carotenoid levels decreased [50]. Maintaining the content of carotenoids in infected *B. juncea* leaves (regardless of day-length regimes), probably, to some extent, balanced the degradation of chlorophylls at the investigated time points (Table S1).

In this study, photosynthetic efficiency was negatively regulated in *B. juncea* plants infected with *A. brassicicola*, similar to the chlorophyll content. A high correlation between the individual parameters of chl *a* fluorescence and the PSII functioning allows for a quick and precise assessment of the photosynthetic apparatus response to abiotic and biotic stresses, including the so-called cross-stress or multistress, caused by the simultaneous influence of more than one stress factor [52,53]. Plants exposed to photoperiodic stress are characterized by a reduced photosynthetic efficiency (determined by F_v_/F_m_ value) compared to the control plants. This highlights the significant role of the circadian rhythm in regulating the function of the photosynthetic apparatus, especially light-dependent reactions. It is particularly interesting in the case of plants additionally exposed to other environmental biotic stresses. Attack of pathogens, such as necrotrophic fungi, usually results in rapid, partial, or complete inactivation of PSII. This is due to inhibition of the electron transfer within PSII or between PSII and photosystem I (PSI), reduction of light energy absorption in LHCII, or disturbance of the energy balance between the supply of assimilation force and the demand for it in the dark phase of photosynthesis [54].

Analysis of the chl *a* fluorescence parameters showed that the photoperiod influenced the degree of PSII damage by the pathogen. A 12 h or longer light period per day caused a significant decrease in the maximum quantum yield of PSII after fungal infection, depending on the time after inoculation and leaf position (age of a leaf). A decrease in the F_v_/F_m_ value caused by *A. brassicicola* has been previously observed in other species of the Brassicaceae family, e.g., *B. oleracea* [35], as well as in *Arabidopsis thaliana* [55]. The observed decrease in photochemical activity in the following hours after inoculation was probably the result of progressive degradation of chloroplasts and disturbance of the thylakoid system, as well as the development of necrotic lesions, which reduced the assimilation area of the leaf. The greatest decreases in F_v_/F_m_ values were observed in plants grown under CL and in the 1st leaf of plants grown under 12 h day/12 h night and 16 h day/8 h night photoperiods (Figure 5). The F_v_/F_m_ values lower than 0.5 indicate severe disturbances in energy transport within PSII in the chloroplasts of cells that have not been directly infected by the fungus and suggest D1 protein degradation or blocked PSII reaction centers by a fungal toxin in infected cells. This is one of the known toxic effects of *A. alternata* in the thylakoid membrane due to competition between plastoquinone QB and the fungal toxin for a binding site in the D1 protein [56]. It is accompanied by a decrease in the pool of open active centers (qL) and a decrease of the qP value, which indicates the blockage of electron transport to the PSII reaction centers and also informs about the proportion of open reaction centers under stressful conditions [57]. However, there is no known *A. brassicicola* toxin that can display such a mode of action [58].

It should be emphasized that exposure to CL itself is a factor limiting the efficiency of the light-dependent processes. This is evidenced by lower mean values of F_v_/F_m_ in the control plants grown under CL compared to the other photoperiods, as well as the increased values of F_0_, which are interpreted as an effect of inhibition of the electron transport between the LHCII and PSII reaction centers [59]. The increase in minimum fluorescence (F_0_) values was especially visible in older leaves (1st) at 48 and 72 hpi in infected plants grown under all the implemented day-length regimes. This shows that photoperiod variation only slightly reduced light absorption by LHCII, but the reduction of this process was more dependent on the pathogen’s activity. An increase in F_0_ is often interpreted as the dissociation of the LHCII core from the PSII reaction center core, which is associated with irreversible damage to the PSII [55].

In many cases, damage to the photosynthetic apparatus caused by *A. brassicicola* or other *Alternaria* species has been correlated with an early increase in heat dissipation to protect PSII from further damage caused by excess excitation energy [54,60]. On the other hand, the PSII response to the stress caused by infection by necrotrophic fungi is also often associated with a rapid decrease in NPQ, both in the necrotic region and in adjacent areas of the leaf blade [35,61]. This is due to insufficient mobilization of the photoprotective mechanisms around the sites of inoculation [62]. In our study, a slight increase in NPQ was detectable only at 24 hpi in the 3rd leaf of plants grown under an 8 h day/16 h night photoperiod. In this case, as in other plant species, the increase in NPQ value took place even before the electron transport efficiency in PSII decreased, as indicated by other parameters of chl *a* fluorescence (e.g., F_v_/F_m_ and Rfd). In plants grown under the other day-length regimes, the NPQ values either did not change (12 h day/12 h night) or decreased (16 h day/8 h night or CL) (Figure 5). A lower level of energy dissipation as heat under the influence of the pathogen in plants grown under a 16 h day/8 h night photoperiod and CL suggests a reduction in the efficiency of the xanthophyll cycle reaction, mainly responsible for NPQ [63,64]. In plants grown under short light periods (less than 12 h), the functioning of the PSII protective mechanisms was not so significantly impaired by the action of the pathogen. This is also confirmed by the values of fluorescence decline ratio in steady-state (Rfd), which gives information about the interaction of the photochemical reactions in the thylakoids with enzymatic reactions of the photosynthesis dark phase [65]. A decrease in the NPQ values in plants grown under a 16 h day/8 h night photoperiod and CL could only be a direct result of the progressive destruction of chloroplasts and not a direct decrease in non-photochemical quenching, but then, considering the F_0_ values, such a decrease should also take place in the case of the other two photoperiods, where it was not observed. In turn, higher energy dissipation by younger leaves (the 3rd leaf) and their higher vitality (Rfd) indicate an attempt to acclimate to the plant’s stressful conditions. The reduction in F_m_ occurred in leaves infected with *A. brassicicola* of plants grown under all the implemented day-length regimes compared to the control. However, in plants grown under 12-h light or more, there was a noticeable tendency for F_m_ to decline on the following days after inoculation (Figure 5). It suggests that electron acceptors in PSII cannot be completely reduced. This trend was not so clearly visible in plants grown under an 8 h day/16 h night photoperiod (Figure 5).

### 3.3. ‘Green Island’ Formation under Prolonged Light Periods

‘Green islands’ surrounding infection sites are best visible in chlorotic/senescing leaves during pathogen infection [26,66], such as dark-green islands (DGIs) in systemically virus-infected leaves [67,68]. In pathogenic fungi-infected leaves, the formation of GIs is mostly initiated in older leaves shortly before senescing, which can be also induced by, e.g., low light or leaf detachment [26]. The GI area is free of any pathogen, with the chlorophyll content slightly reduced or similar as in uninfected tissues, and photosynthetic activity is also maintained [28,66]. It is thought that the polyamines and cytokinins levels are involved in GI formation and delaying senescence in this region of the leaf. However, the physiology of the necrotrophic fungi-induced GIs may differ from the GIs formed during infection with biotrophic fungi. Although, it has to be emphasized that investigations concerning GI formation are very limited [26].

In brassicas, GIs have been observed in *B. napus* infected with *L. maculans* and *A. brassicae* [26]. The formation of GIs in *B. juncea* has been described in detached cotyledons during *A. candida* infection [69] and in leaves of mature *B. juncea* plants naturally infected with *A. brassicicola* under field conditions [70]. Interestingly, cytokinin-like substances have been discovered in the area of GIs in infected *B. juncea* with *A. brassicicola* [70]. An elevated cytokinin level is also required for defense against high light and altered photoperiod stresses [71]. In this study, GIs were formed in susceptible *B. juncea* in response to *A. brassicicola* infection in the oldest leaves and were visible on a leaf blade at the background of chlorotic, yellowing tissues only in plants grown under a long-day photoperiod (16 h day/8 h night) or CL (Figure 6). The appearance of a GI phenotype and chlorosis a day earlier (at 48 hpi instead of 72 hpi) in *B. juncea* inoculated on the 35th day of growth compared to 28-day-old inoculated plants indicate that plant age or leaf age play an important role in the induction of GIs and acceleration of senescence in response to *A. brassicicola* infection.

At the initial stages of *B. oleracea* infection, *A. brassicicola* induces gradual changes in the chloroplast ultrastructure, depending on the distance from the infecting hyphae. Most frequently, chloroplasts changed their shape from lenticular to round, disintegrate their envelope and stroma, as well as observe the disappearance of grana and damaged thylakoids [35]. Similarly, damaged chloroplasts with large starch grains or their hydrolyzed remains have been previously described at infection sites of *B. juncea* leaves infected with *A. brassicicola* [19], as well as in this study (Figure 8). However, chloroplasts became irregular in shape, with disturbed thylakoids and many plastoglobules in the GIs at 48 hpi (Figure 8k). An increased number of plastoglobules in the chloroplast of GI cells also have been observed in *B. juncea* infected with *A. candida* [66]. To summarize, further studies on the nature of *A. brassicicola*-induced GIs are required.

## 4. Materials and Methods

### 4.1. Plant Material and Plant Growth Conditions

Seeds of Indian mustard (*Brassica juncea* [L.] Czern.) were planted and grown in a commercial soil:perlite mixture (15:1), pH 5.8–6.2, in a plant growth room under controlled conditions: temperature 22 °C ± 2 °C, relative humidity of 65%, and fluorescent light intensity at least 120 μmol m^−2^ s^−1^ (Super TLD Philips 865) [19]. To avoid light/dark stress and an adaptation period, the plants were subjected to four different day-length regimes: 8 h day/16 h night, 12 h day/12 h night, 16 h day/8 h night, and continuous light (24 h day/0 h night) during a whole growing period and all experiments were conducted simultaneously. Plants were grown to a stage of four fully developed leaves for approximately 28 days or for 35 days when ‘green island’ formation was examined. 

To compare the leaf size parameters under different day-length regimes, the 1st (the oldest) and the 3rd leaves were detached, and the leaf area and width in the x-axis were measured using the WinDIAS_3 Leaf Image Analysis system (Delta-T Devices, Cambridge, UK). Measurements were performed during necrosis development experiments. The means ± min/max were calculated from 16–20 measurements of each leaf per photoperiod.

### 4.2. Pathogen Growth and Inoculum Preparation

The necrotrophic fungus *Alternaria brassicicola* (ATCC 96836) was grown on potato dextrose agar plates (Difco, the Netherlands) at 22 °C ± 2 °C in the dark for 7–10 days. The conidial suspension was prepared by floating the fungal culture with sterile distilled water and re-suspending it to a concentration of 5 × 10^5^ conidia per mL [72]. The conidial suspension as prepared was used in all experiments.

### 4.3. Inoculation Procedure and Samples Harvesting

The 1st and 3rd leaf of each plant was inoculated with two or four 10 µL drops of conidial suspension (5 × 10^5^ conidia per mL of distilled water) or in the case of the control plants with drops of distilled water. Plants were inoculated during light periods, approximately 4–6 h after the night period (around 10:00–12:00 a.m. local time). The inoculated plants were incubated in translucent boxes to maintain a high humidity under the same light and temperature conditions as described above. All experiments were conducted simultaneously under different day-length regimes.

To investigate the GI phenotype, plants were grown under a 16 h day/8 h night photoperiod and CL simultaneously. Four mature leaves of plants, from the oldest (1st) to the youngest (4th) leaf, were inoculated with conidial suspension. The experiment was repeated independently twice.

The samples for analysis of photosynthetic pigment contents were harvested 4–6 h after switching on the light (about 10:00–12:00 a.m. local time) and stored at −70 °C until used. Chlorophyll *a* fluorescence measurements and collection of samples for transmission electron microscopy analyses also were conducted at the same time of day.

### 4.4. Disease Development

The spread of visible necrosis was evaluated every 24 h for 3 days and measurements of necrosis diameters were performed with a caliper. Six plants per experiment were inoculated. Each experiment was repeated independently 4 times (*n* = 4).

### 4.5. Photosynthetic Pigments Content

Samples of control and infected leaves were extracted in 100% methanol and measured using a spectrophotometer PowerWave XP (BioTek, Winooski, VT, USA). Chlorophyll *a*, *b*, chlorophyll *a*:*b* ratio, carotenoids, and total chlorophyll:carotenoids ratio were calculated according to Wellburn [73]. Three control and three inoculated plants were used per time point of each photoperiod exposure. The experiment was repeated independently four times (*n* = 4). Detailed statistical analysis of the chlorophyll and carotenoid contents is available in Supplementary Material Tables S1 and S2 and Figure S2.

### 4.6. Chlorophyll a Fluorescence

Plants were dark-adapted for 30 min, and the 1st and 3rd leaves were detached and subjected to measurements of chlorophyll *a* fluorescence quenching using a Handy FluorCam 1000-H System (Photon Systems Instruments, Drasov, Czech Republic) according to the manufacturer’s built-in protocol. The whole procedure was performed as described by Macioszek et al. [35]. In each experiment, 3–6 control and 3–6 inoculated plants per time point of each photoperiod were analyzed. The experiment was repeated independently 3 times (*n* = 3). Minimum fluorescence (F_0_) and maximum fluorescence (F_m_) in the dark-adapted state were measured and maximum quantum yield of PSII (F_v_/F_m_), non-photochemical quenching (NPQ), fluorescence decline ratio (Rfd), coefficient of photochemical quenching (qP), and the fraction of PSII open centers (qL) were calculated for a steady-state light period. Statistical analysis of the parameters is available in Supplementary Material Tables S3 and S4 and Figure S3.

### 4.7. Transmission Electron Microscopy (TEM)

The samples were dissected from the control and the infected 1st and 3rd leaves at 24 and 48 hpi. Following morphological changes occurring on leaves during the infection development, they were collected from the regions of necrosis, edge of necrosis, and outside necrosis for light and transmission electron microscopy examinations. In the case of the 1st leaf at 48 hpi, the edge of the necrosis corresponds to the greenish area (‘green island’) and the area outside the necrosis corresponds to chlorosis (Figure 6a,d). The samples were fixed in 3% (*v*/*v*) glutaraldehyde in a 0.1 M cacodylate buffer (pH 6.8) at 4 °C for 2 h and then post-fixed in 2% (*w*/*v*) osmium tetroxide for 2 h. Then the samples were dehydrated in an ethanol series and propylene oxide and embedded in Epon–Spurr resin [35]. The semi-thin (1 µm thick) and ultra-thin (80 nm thick) sections were obtained using a Reichert Jung microtome (Leica, Wetzlar, Germay). The semi-thin sections were analyzed using a Nikon light microscope (Nikon, Tokyo, Japan). The ultra-thin sections were stained with uranyl acetate and lead citrate and examined with a Jeol 1010 TEM (Jeol, Tokyo, Japan) operating at 80 kV. The representative TEM images were selected from 20–30 pictures taken of each leaf blade region.

### 4.8. Statistical Analysis

The statistical analyses of all the obtained data were performed using analysis of variance (ANOVA) and a post-hoc Duncan’s test (*p* < 0.05) using STATISTICA v.13.3. software (Tibco Software Inc., StatSoft, Krakow, Poland)

The charts and heatmaps were prepared using GraphPad Prism (https://www.graphpad.com, accessed on 9 October 2020) or MS Office Excel Software. All figures were composed using Adobe Photoshop or Corel Software.

## 5. Conclusions

Infection of *Brassica* crop plants by a necrotrophic fungus, *A. brassicicola*, causes yield losses worldwide. The current study showed that development of black spot disease in susceptible *B. juncea* is leaf position- and day length-dependent. Although, necrosis progression was significantly inhibited in plants grown under CL, especially in the 1st, older leaf. Light/dark periods (short or long days) differentially decreased the content of chlorophylls and negatively regulated the light-dependent reactions of photosynthesis, inhibiting the performance of PSII in infected leaves. In the oldest leaves of plants grown under a 16 h day/8 h night photoperiod and CL, the GI phenotype and accelerated senescence appeared in response to the *A. brassicicola* infection. Ultrastructural analysis of the chloroplasts from different regions of infected leaves revealed that at least at the initial stages of formation the GI could be photosynthetically active. However, the disappearance of the greenish area of the GI, senescence, and yellowing of the whole blade of the oldest leaves were observed at later time points of post-inoculation. These results provide new insights into the development of black spot disease in susceptible *B. juncea* plants under various environmental conditions. This is important both for a better understanding of the *A. brassicicola* pathogenicity and for the protection of crops against this pathogen. The complexity of the problem requires further research on cross-stress in *Brassicaceae* plants.

## Data Availability

Data is contained within the article and Supplementary Materials.

## Supplementary Materials

The following are available online at https://www.mdpi.com/article/10.3390/ijms22168435/s1.
