# Supplementary material for: The Effect of Photoperiod on Necrosis Development, Photosynthetic Efficiency and ‘Green Islands’ Formation in Brassica juncea Infected with Alternaria brassicicola
Source: Int J Mol Sci. 2021 Aug 5;22(16):8435. doi: 10.3390/ijms22168435 (PMC8395102; doi:10.3390/ijms22168435)
Supplement: Supplementary file 1 [file ijms-22-08435-s001.zip › Suplementary file Figure S1-S4.pdf]

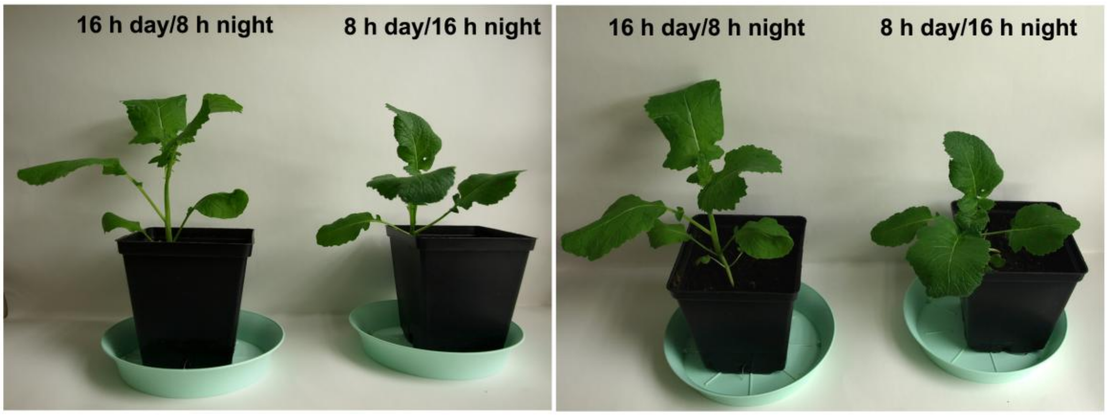

Figure S1. *Brassica juncea* plants grown under 16 h day/8 h night and 8 h day/16 h night photoperiods. The plants grown under an 8 h day/16 h night photoperiod had shorter stems than plants grown under a 16 h day/8 h night photoperiod.

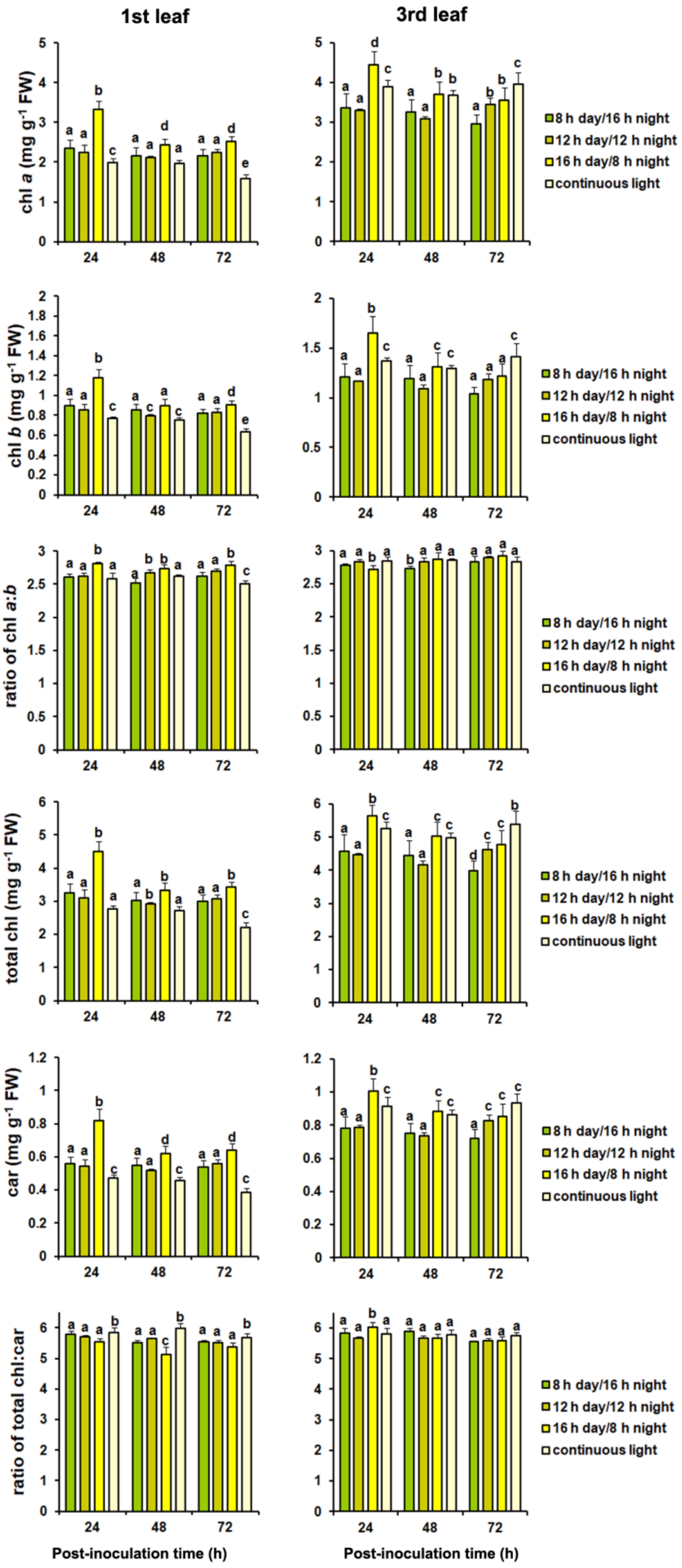

Figure S2. Influence of day length on chlorophyll and carotenoid contents in control leaves of *Brassica juncea*. The means ± SE were obtained in 4 independent experiments (n = 4). Different letters indicate statistical difference between means within leaf position according to Duncan`s test (p<0.05). Abbreviations: car – carotenoids, chl – chlorophyll.

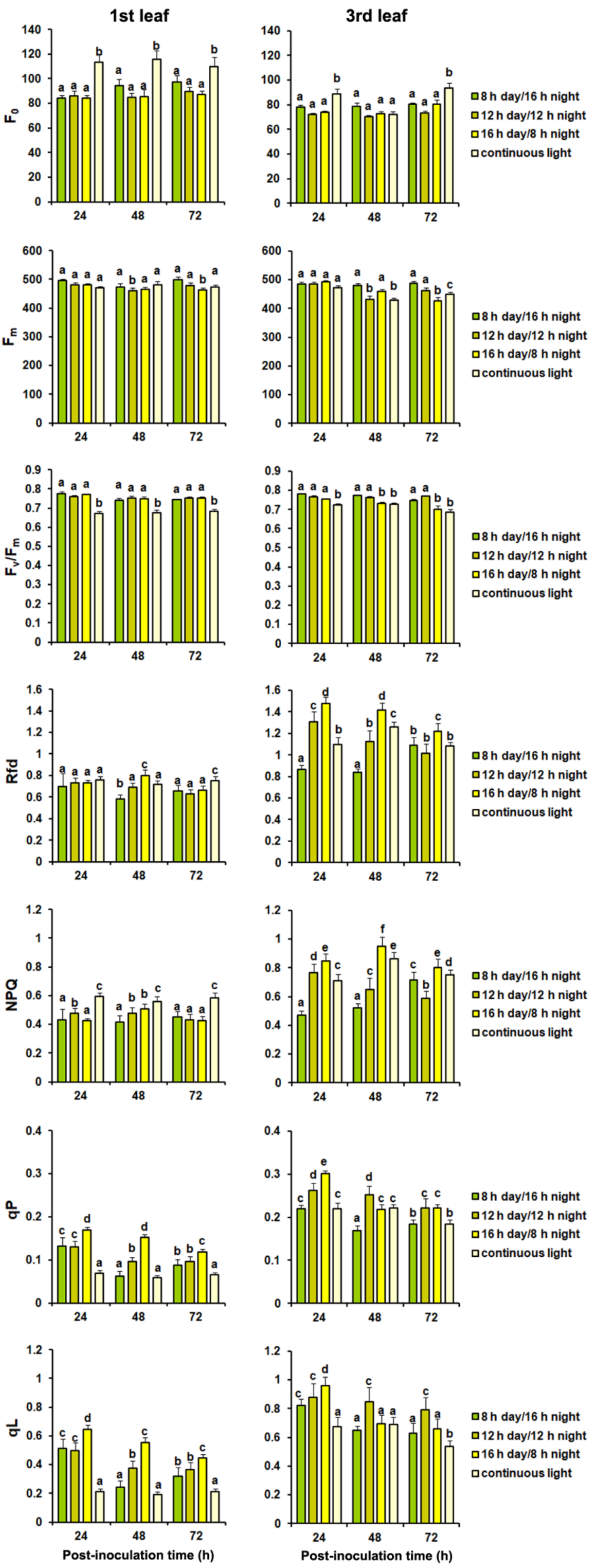

Figure S3. Influence of day length on chlorophyll *a* fluorescence parameters in control leaves of *Brassica juncea*. The means ± SE were obtained from 3 independent experiments (n = 3). Different letters indicate statistical difference between means within leaf position according to Duncan`s test ($p < 0.05$).

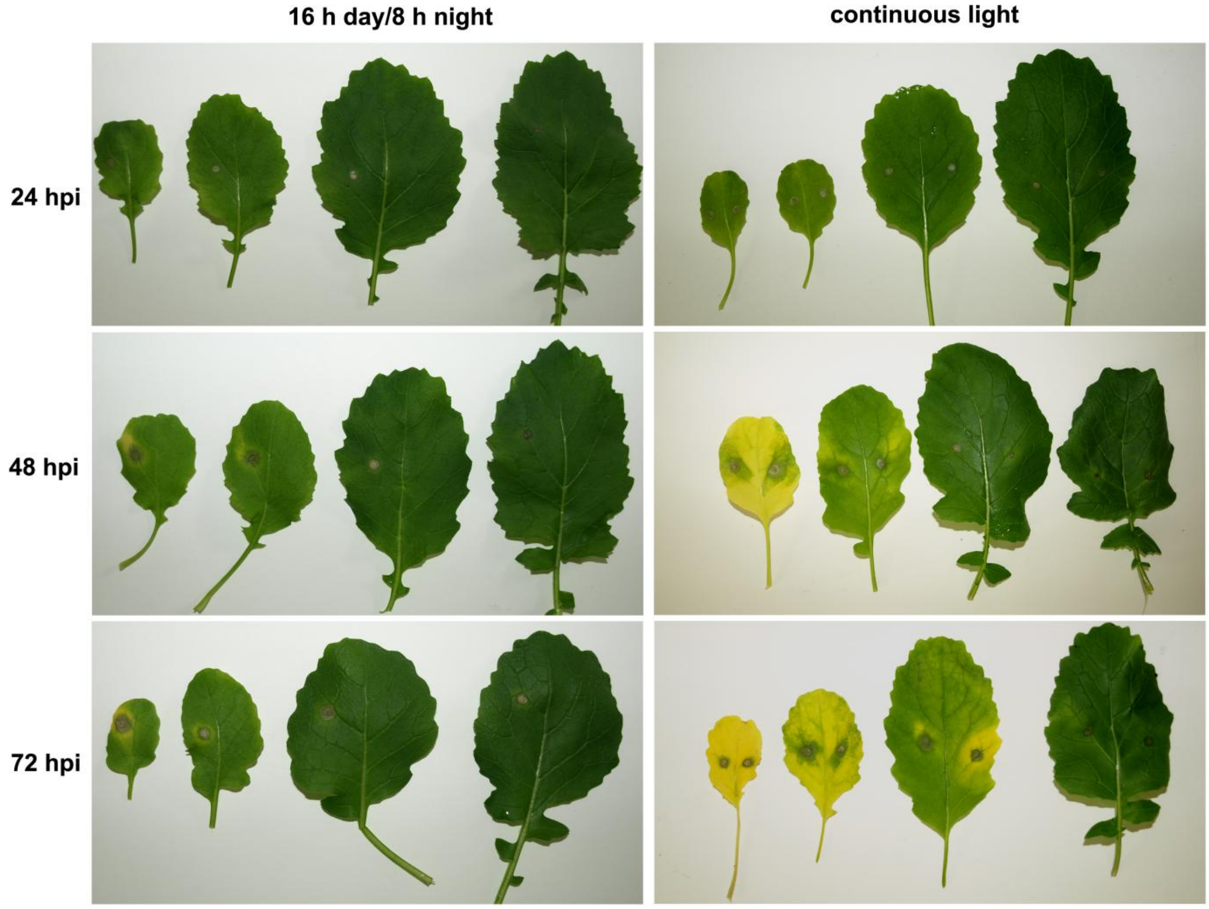

Figure S4. Time-course formation of 'green island' phenotype in *B. juncea* plants during infection with *A. brassicicola* under a 16 h day/8 h night photoperiod and continuous light.
